# 

**Published:** 1886-09-20

**Authors:** 


### TABLE OF CONTENTS

ORIGINAL AND SELECTED ARTICLES.

A Case of Obstructed Bowel, by R. G. Roy, M.D., Professor Materia Medica in Southern Medical College....325
The Rational Treatment of Nasal Catarrh, by A. G. Hobbs, M. D., Atlanta, Ga., Professor of Diseases of the Eye, Ear and Throat in Southern Medical College....328
A Diagnosis, by H. Christopher, M. D., of Mo...330
Observations on Diabetes Mellitus, by William Bailey, A.M., M.D., Louisville....334
Detroit Gynæcological Society....337
Administration of Salicylates; A Pasteur Institute in New York....340

SOCIETY REPORTS.

Excision of Anal Circumference with Hemorrhoids....341
White Swelling of the Knee....343

ABSTRACTS AND GLEANINGS.

Marvelous Results of a New Anæsthetic....344
The Local Use of Liquid Ergot, Normal, in Chronic Gonorrhœa....345
The Preventive Treatment of Syphilis....346
Removal of Foreign Bodies from the Ear....347
Ethoxycaffeine in the Treatment of Migraine; On the Statistics of 3036 Cases of Labor, by Hiram Corson, M. D....348
Therapeutic Value of Coca Preparations in Children....350
Fatal Results from "Splitting the Cervix;" Water as a Diuretic....351
Permanganate Potasium in Snake Bites....352
Puncture of the Nerve Sheath in Sciatica; Salicylate of Cocaine in Asthma....353
The Use of Hot Water in some of the Corneal and Conjunctival Inflammations; Turpine in Chronic Bronchitis....354

SCIENTIFIC ITEMS.

The Latest Microscopical Discoveries; Danger from Umbrellas at Sea; A Cigar....355
Thunder Storms....356

PRACTICAL NOTES AND FORMULÆ.

For Vaginismus; Cough....357
For Intermittent Fever; For Rheumatism; For Gonorrhœa....358

EDITORIALS AND MISCELLANEOUS.

Editorial Notices....359
Our Trip to Salt Spring....360
An Improved Method of Medication....362
Books and Pamphlets Received....363
Special Notices....364

